# Is Anterior Longitudinal Ligament Rupture During Posterior Corrective Surgery for Adult Spinal Deformity a Phenomenon Unique to When Combined with Lateral Lumbar Interbody Fusion? -Finite Element Analysis with Comparison to When Combined with Posterior Lumbar Interbody Fusion-

**DOI:** 10.3390/jcm14217460

**Published:** 2025-10-22

**Authors:** Takaya Imai, Hiroki Takeda, Yuichiro Abe, Koutaro Kageshima, Yuki Akaike, Soya Kawabata, Nobuyuki Fujita, Shinjiro Kaneko

**Affiliations:** 1 Department of Spine and Spinal Cord Surgery, School of Medicine, Fujita Health University, 1-98 Dengakugakubo, Kutsukake-cho, Toyoake 470-1192, Aichi, Japan; tkyaaa0405@gmail.com (T.I.); htakeda@fujita-hu.ac.jp (H.T.); menchi@athena.ocn.ne.jp (Y.A.); 2 Department of Orthopedic Surgery, School of Medicine, Fujita Health University, Toyoake 470-1192, Aichi, Japan; koutaro.kage.98@gmail.com (K.K.); nineteamjump@gmail.com (Y.A.); fb_kawabata@yahoo.co.jp (S.K.); nfujita2007@gmail.com (N.F.)

**Keywords:** scoliosis, kyphosis, anterior longitudinal ligament, surgical procedure, finite element analysis

## Abstract

**Background**: The occurrence of ALL rupture during posterior correction of adult spinal deformity (ASD) was rare before the introduction of lateral lumbar interbody fusion (LLIF) but has become more frequent recently. It remains unclear whether this phenomenon is unique to LLIF-combined procedures or primarily related to enhanced corrective ability. **Methods**: The research method used in this study is finite element analysis (FEA). Using preoperative computed tomography images, LLIF cage (L group) or posterior lumbar interbody fusion (PLIF) cage (P group) were placed in the disc space with identical lordotic angles and distances from the anterior vertebral body edge for the same patients’ samples. Finite element simulations of corrective procedures were conducted. A spring simulating the ALL was introduced into the FEA, and the load on the ALL was evaluated with either LLIF or PLIF cage placement. Spring elongation directly measured the load on the ALL, while the location of the rotation center served as an indirect evaluation. Two different types of corrective procedures were created, one of which is mimicking ASD correction. For both procedures, the load to ALL was measured using abovementioned parameters when either LLIF cage (L group) or PLIF cage (P group) was used. The load to ALL was compared between L group and P group. **Results**: The degree of spring elongation during the simulation of a corrective procedure significantly decreased in the L group compared to the P group only in the model which is mimicking ASD correction (*p* = 0.006, Cohen’s d = 2.33, Power (1−β) = 0.956). The rotation center was significantly more posteriorly located in the P group than that in the L group in both models. These differences were more obvious in the model mimicking ASD correction (*p* = 0.0013, Cohen’s d = 2.00, Power (1−β) = 0.891). **Conclusions**: Our findings suggest that the use of a PLIF cage, which has a longer anterior–posterior cage length, caused the posterior edge of the cage to act as a pivot point. This configuration places greater leverage on the ALL, potentially leading to rupture during posterior correction procedures. This phenomenon, ALL rupture during posterior correction for ASD, is thought to be associated with increased corrective capabilities rather than being specific to the geometry of the LLIF cage.

## 1. Introduction

In recent years, lateral lumbar interbody fusion (LLIF), including oblique lateral interbody fusion (OLIF) [1] and extreme lateral interbody fusion (XLIF) [2], has gained widespread acceptance in the surgical management of adult spinal deformity (ASD) (degenerative lumbar scoliosis, kyphosis and kyphoscoliosis, etc.) and lumbar degenerative diseases [1,2,3,4,5,6,7,8,9,10]. LLIF offers several advantages in these contexts, notably robust bony fusion facilitated by extensive contact between the LLIF cage and the adjacent vertebral body, including its cortical portion. Literature has consistently reported high fusion rates with LLIF (93–98%) [1,2,3,4,5,6,7,8,9,10].

Grade 2 posterior column osteotomy (PCO) is a highly effective procedure in ASD surgery for obtaining lumbar lordosis [11,12,13]. Furthermore, no matter what type of lumbar degenerative disease, obtaining proper lumbar lordosis is essential when fusing any segment to achieve long-term surgical goals, as patients lose compensatory ability within the fusion area postoperatively. Meanwhile, grade 2 PCO is the procedure that potentially increases the risk of pseudoarthrosis due to bilateral facet joint removal. Before LLIF introduction, grade 2 PCO was commonly combined with posterior or transforaminal lumbar interbody fusion (PLIF or TLIF), which provide less contact area between the cage and adjacent vertebrae compared to LLIF. Enhanced bone fusion potential with LLIF may allow for substantial lumbar lordosis correction through multilevel grade 2 PCO in ASD cases with less the risk of pseudoarthrosis. We have previously demonstrated that the combination of OLIF and grade 2 PCO is effective for achieving sufficient lumbar lordosis and robust bone fusion in ASD, as well as other lumbar degenerative conditions [13]. We have applied this OLIF and grade 2 PCO procedure extensively and observed a high rate of circumferential bone fusion postoperatively [13].

**Figure 1:**
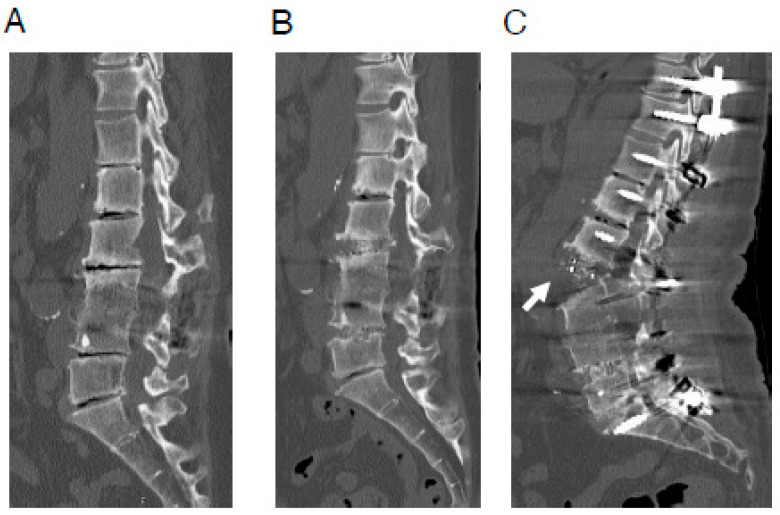
A representative case of ALL rupture during posterior correction procedure. A 64-year-old woman with adult spinal deformity, who had undergone postero-lateral fusion at the L3/4 level previously at another hospital. (**A**) Preoperative CT image. (**B**) CT image after OLIF (L2/3·L4/5). (**C**) CT image after posterior correction procedure. In this case, surgery was performed using less lordotic (6°) OLIF cages, which were most lordotic cage available at the time. Anterior longitudinal ligament rupture (white arrow) was observed after the posterior correction procedure. ALL—anterior longitudinal ligament; CT—computed tomography; LLIF—lateral lumbar interbody fusion; OLIF—oblique lateral lumbar interbody fusion. Reproduced with permission from Takeda, H.; Abe, Y.; Imai, T.; Rashid, M.Z.M.; Ikeda, D.; Kawabata, S.; Nagai, S.; Hachiya, K.; Fujita, N.; Kaneko, S. Elucidation of the mechanism of occasional anterior longitudinal ligament rupture with posterior correction procedure for adult spinal deformity using LLIF-finite element analysis of the impact of the lordotic angle of intervertebral LLIF cage. *Medicina* 2023, *59*, 1569; published by MDPI, 2023. [32].

However, we currently have several unresolved issues. One of the notable current issues is the occasional rupture of the anterior longitudinal ligament (ALL) observed during posterior corrective procedures for ASD using LLIF (Figure 1) [14,15,16]. As ALL rupture increases the risk of pseudoarthrosis, preventing it is paramount. However, the exact mechanism of ALL rupture during posterior corrective procedures remains unclear. Finite element analysis (FEA) is a computational method originally developed in industrial sectors, such as construction, and subsequently applied in biomechanical studies in the medical field to simulate mechanical scenarios using computer models [17,18,19,20,21,22,23,24,25,26,27,28,29,30,31]. We have employed FEA to investigate the mechanism of ALL rupture during posterior corrective procedures from various perspectives [32].

It is notable that ALL rupture during posterior corrective procedure is a phenomenon that was infrequent prior to the introduction of LLIF. Previously, corrective surgery for ASD was performed using only PLIF or TLIF for lumbar intervertebral body fusion [1,2,3,4,5,6,7,8,9,10]. However, as the corrective ability for ASD has improved recently from various perspectives, it remains unclear whether ALL rupture during posterior correction is unique to LLIF-combined procedures or is associated with overall improvements in corrective capabilities. In this study, we aimed to investigate this research question using FEA. Therefore, the purpose of this study is to clarify whether ALL rupture during posterior correction is unique to LLIF-combined procedures or is associated with overall improvements in corrective capabilities.

## 2. Materials and Methods

### 2.1. Establishment of the Three-Dimensional (3D) Finite Element (FE) Model of the Lumbar Spine

This research is a basic study based on CT data obtained in clinical settings. Clinical data were collected retrospectively. Overall, this study was conducted from October 2020 to March 2025. Five patients (three males and two females; average age, 63.0 ± 7.3 years old) with degenerative lumbar disease who underwent CT scans for surgical planning were included in the analysis. All of these patients were diagnosed with lumbar canal stenosis and underwent lumbar spine surgery at our institution. Cases with severe spinal deformities such as scoliosis were not included in order to simplify the FE model so that various factors would not be included. All of these patients had moderate to severe spinal canal stenosis on imaging. Additionally, these patients had symptoms thought to be due to spinal canal stenosis that were interfering with their daily lives. Furthermore, these patients had been thoroughly treated with conservative therapy, but because the effectiveness of conservative therapy was limited, their attending physicians deemed them suitable for surgery.

In this analysis using FEA, it is necessary to use plain CT scans rather than post-myelography CT scans. In our usual protocol, we perform post-myelography CT scans for lumbar degenerative disease patients who are scheduled for surgery, so plain CT scans are only performed in limited cases, such as when the patient is allergic to contrast agents. There were not many cases that met these conditions, and taking into account the characteristic of research using FEA that results do not vary significantly between cases, five cases were included in this analysis. Each patient’s custom spinal FE model was constructed using preoperative CT Digital Imaging and Communications in Medicine (DICOM) data [30,32]. ANSYS Workbench 2020 (ANSYS Japan, Tokyo, Japan) software was employed for modeling the lumbar spine and conducting surgical simulations. DICOM data were imported into Mimics Research 23.0 (Materialize, Leuven, Belgium) software to generate 3D vertebral models. The FEA procedures followed previous literature methodologies [32]. The basic FEA method of this study is almost identical to that of our previous paper, so we will not go into detail here as there would be a lot of overlap with that paper [32]. Condition settings that are not described here are the same as those in our previous paper [32].

**Figure 2:**
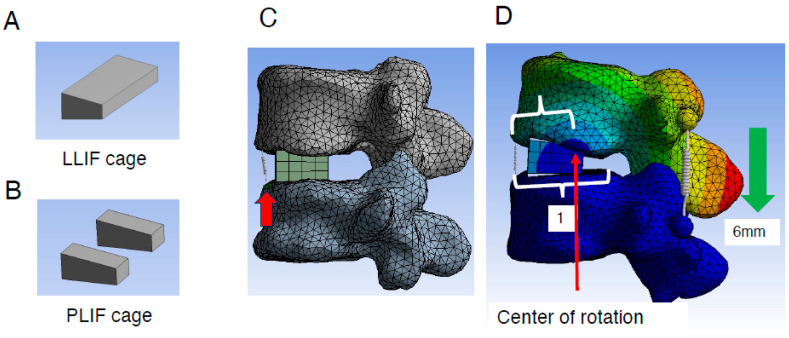
Creation of lumbar FE model and placement of the LLIF/PLIF cage. (**A**,**B**), Geometry of the LLIF (**A**) and PLIF cages (**B**) placed into the L4/5 intervertebral space. (**C**), LLIF cages with a height of 10 mm and A-P length of 18 mm were added. To assess the extent of ALL burden during different types of correction procedures, a spring (red arrow) was introduced to mimic ALL at its location (details are described in Section 2). (**D**), Evaluation method of the rotation center during correction procedures (details are described in Section 2). ALL indicates anterior longitudinal ligament; FE, finite element; LLIF, lateral lumbar interbody fusion; PLIF, posterior lumbar interbody fusion.

Once the 3D models were imported into ANSYS Workbench 23.0, FE meshing was performed, and either a single LLIF cage (10 mm height, 18 mm anterior–posterior (A-P) length) or two PLIF cages (each 10 mm height, 22 mm A-P length) were added (Figure 2A,B). Both the LLIF and PLIF cages were set at a lordotic angle of 12°.

The L4 caudal endplate and L5 cranial endplate were aligned parallel to each other, with the anterior edges of the LLIF or PLIF cages positioned 3 mm posterior to the anterior edge of the L5 cranial endplate. Bilateral and symmetrical placement of two PLIF cages was performed. To assess the burden on the ALL during different correction procedures, a spring model representing the ALL was introduced at its anatomical location (Figure 2C). The elongation length of the spring during the correction procedure was measured to quantify the burden on the ALL. Additionally, the location of the rotation center was analyzed. This was calculated by dividing the distance between the anterior edge of the L5 cranial endplate and the rotation center (Figure 2D) by the total distance between the anterior and posterior edges (Figure 2D).

The spring constant was set to 100 N/mm, and a coefficient of friction of 0.5 was applied between the endplate and the LLIF or PLIF cage, based on similar conditions reported in previous studies [31]. The model did not differentiate between cortical and cancellous bone but was designed to standardize the simulation across all elements, ensuring bone fragility did not influence the results.

### 2.2. Construction of Two Different Types of FE Models and Comparison Between the Usage of LLIF Cage or PLIF Cage for Each Procedure

**Figure 3:**
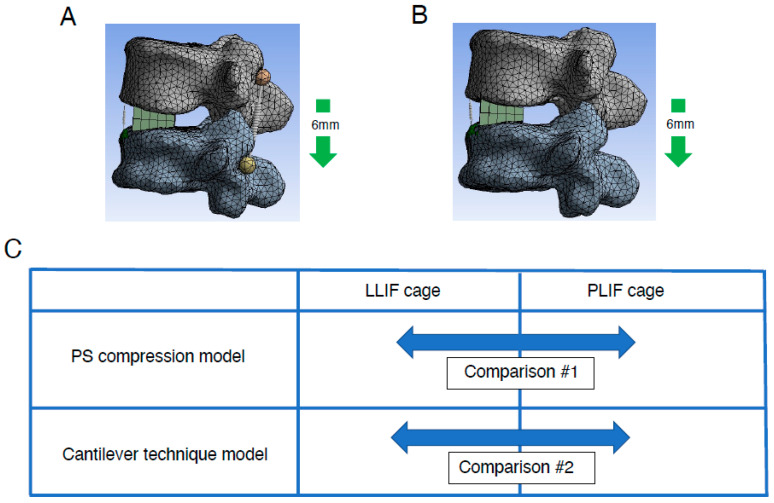
Creation of lumbar FE model with two different types of correction procedures. Lumbar FE model with two different types of correction procedures: PS compression model (**A**) and spinous process displacement model (cantilever technique model) (**B**) was created (details are described in Section 2). (**C**), Comparison between two deferent types of intervertebral cages, LLIF cage and PLIF cage, in two different types of FEA models. Both types of intervertebral cages were analyzed using PS compression models and the cantilever technique model. FE indicates finite element; LLIF, lateral lumbar interbody fusion; PLIF, posterior lumbar interbody fusion; PS, pedicle screw.

ALL rupture is occasionally observed during correction surgery for ASD but not during fusion surgery for common degenerative lumbar diseases [14]. Accordingly, we constructed two different types of FE models as previously described [32]. In fusion surgery for common lumbar degenerative disease, compression force is applied between the pedicle screws (PSs) to stabilize the cage placed between the vertebral bodies and to create lumbar lordosis. To simulate fusion surgery for common degenerative lumbar diseases, we created an FE model incorporating PSs (PS compression model). The L5 vertebral body was restrained, and a 5.5 mm diameter and 45 mm length PS was inserted into L4 and L5. The Young’s modulus was set at 12,000 MPa for the vertebral body, LLIF or PLIF cage, and PS, with a Poisson’s ratio of 0.3. On both the left and right sides, a spring connected between the heads of the L4 and L5 PSs. To simulate the compression procedure in fusion surgery for common degenerative lumbar diseases, the distance between the L4 and L5 heads of the PSs was reduced by 6 mm. The elongation degree of the spring at the ALL site and the location of the rotation center were evaluated (Figure 3A).

Currently, the correction method for ASD is mainly translation for correction of the coronal plane and the cantilever technique for correction of the sagittal plane. The reason behind this is that the tools and implants used for correction have been significantly improved. As described, the cantilever technique is a surgical technique mainly used to correct the sagittal plane. It is a basic reduction technique for kyphotic correction since the discovery of the PS several decades ago. The principle of this technique is achieving reduction by securing a pre-bend rod to PSs distal or proximal to apex of deformity following osteotomy procedure. On the other hand, it is not realistic to reproduce the cantilever technique in detail in an FEA model. Therefore, we also constructed a spinous process displacement model (cantilever technique model) to simulate the cantilever technique as previously described [32]. Because the point at which force is applied to create lumbar lordosis is more dorsal, this model uses a model that shortens the distance between the spinous processes, which are the most dorsal of the spinal components, as a model that mimics the cantilever technique. Briefly, under the restraint condition of the caudal (L5) vertebral body, a force was applied to displace the spinous process of rostral level (L4) by 6 mm in the minus *Z*-axis direction. The elongation degree of the spring at the ALL site and the location of the rotation center (Figure 3B) were evaluated. Using the same method as in the PS compression model, the elongation degree of the spring at the ALL site and the location of the rotation center were evaluated in the spinous process displacement model (cantilever technique model).

Analyses using models for these two different correction methods were performed on the five cases mentioned above. Therefore, the analyses using the two different correction methods were performed using the same five cases.

Comparisons were made between conditions with the usage of either LLIF cage or PLIF cages in each model: the PS compression model and the cantilever technique model (Figure 3C). In each model, simulations were performed incorporating grade 2 PCO.

### 2.3. Statistical Analyses

IBM SPSS Statistics (version 27; IBM Corp., Armonk, NY, USA), G*power 3.1 (free application available on the internet) [33,34] and Microsoft Excel (version 16.89.1; Microsoft Corp., Redmond, WA, USA) were used for statistical analyses. The level of significance was set at *p* < 0.05. Student’s *t*-test was used for all the statistical analyses between two groups. Cohen’s d was calculated using Microsoft Excel and Power (1−β) was calculated using G*power 3.1 [33,34].

## 3. Results

### 3.1. Assessment of the Elongation Degree of ALL in the PS Compression Model

**Figure 4:**
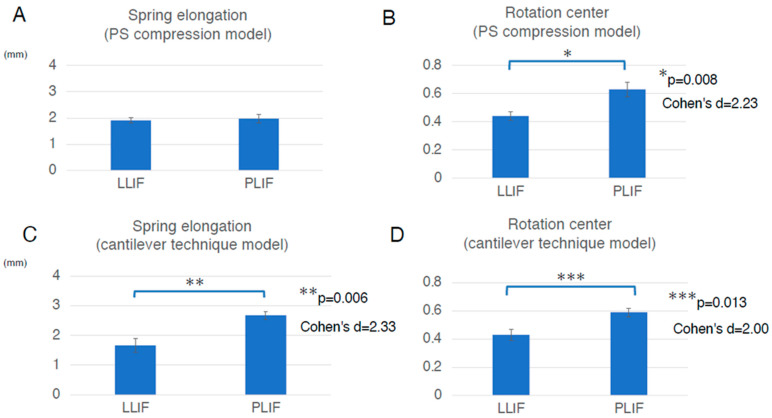
Assessment in two different types of the FEA model, the PS compression model and the cantilever technique model. (**A**), In the PS compression model, the average spring elongation was 2.0 ± 0.4 mm in the PLIF group and 1.9 ± 0.2 mm in the LLIF group (average ± standard deviation (SD)). Although the difference between the two groups was not statistically significant, the elongation in the PLIF group tended to be larger than that in the LLIF group (*p* = 0.773). (**B**), In the PS compression model, the average location of the rotation center was 0.63 ± 0.10 in the PLIF group and 0.44 ± 0.06 in the LLIF group (average ± SD). The rotation center in the PLIF group was significantly more posterior than that in the LLIF group (*p* = 0.008, Cohen’s d = 2.23, Power (1−β) = 0.941). The values for Cohen’s d and Power (1−β) were large enough to ensure the magnitude of the significant difference and the validity of the test method. (**C**), In the cantilever technique model, the average spring elongation in the PLIF and LLIF groups was 2.7 ± 0.3 mm and 1.7 ± 0.5 mm, respectively (average ± SD). The elongation degree in the PLIF group was significantly larger than that in the LLIF group (*p* = 0.006, Cohen’s d = 2.33, Power (1−β) = 0.956). The values for Cohen’s d and Power (1−β) were large enough to ensure the magnitude of the significant difference and the validity of the test method. (**D**), In the cantilever technique model, the average location of the rotation center in the PLIF group was 0.59 ± 0.06, while that in the LLIF group was 0.43 ± 0.10 (average ± SD). The location of the rotation center in the PLIF group was located significantly more posterior than that in the LLIF group (*p* = 0.013, Cohen’s d = 2.00, Power (1−β) = 0.891). The values for Cohen’s d and Power (1−β) were large enough to ensure the magnitude of the significant difference and the validity of the test method. Bar graphs show the mean and standard error. FEA indicates finite element analysis; LLIF, lateral lumbar interbody fusion; PLIF, posterior lumbar interbody fusion; PS, pedicle screw.

**Figure 5:**
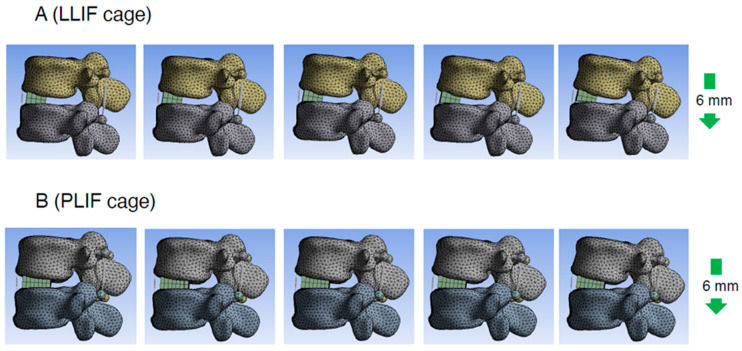
Representative cases of the PS compression model. Consecutive images with compression process in PS compression model (right side is later phase). LLIF indicates lateral lumbar interbody fusion; PLIF, posterior lumbar interbody fusion; PS, pedicle screw.

To assess the direct burden on the ALL in the PS compression model, the elongation degree of the spring placed at the ALL location was evaluated. The average spring elongation was 2.0 ± 0.4 mm in the PLIF group and 1.9 ± 0.2 mm in the LLIF group (average ± standard deviation (SD)). Although the difference between the two groups was not statistically significant, the elongation in the PLIF group tended to be larger than that in the LLIF group (*p* = 0.773) (Figure 4A and Table 1). Representative cases of the PS compression model are shown in Figure 5.

### 3.2. Assessment of the Rotation Center Location in the PS Compression Model

To assess the indirect burden on ALL in the PS compression model, the location of the rotation center was also evaluated. The average location of the rotation center was 0.63 ± 0.10 in the PLIF group and 0.44 ± 0.06 in the LLIF group (average ± SD). The rotation center in the PLIF group was significantly more posterior than that in the LLIF group (*p* = 0.008, Cohen’s d = 2.23, Power (1−β) = 0.941) (Figure 4B and Table 1). The values for Cohen’s d and Power (1−β) were large enough to ensure the magnitude of the significant difference and the validity of the test method. Therefore, the indirect burden on the ALL in the PLIF group in the PS compression model was significantly larger than that in the LLIF group.

### 3.3. Assessment of the Elongation of ALL in the Cantilever Technique Model (Spinous Process Displacement Model)

**Figure 6:**
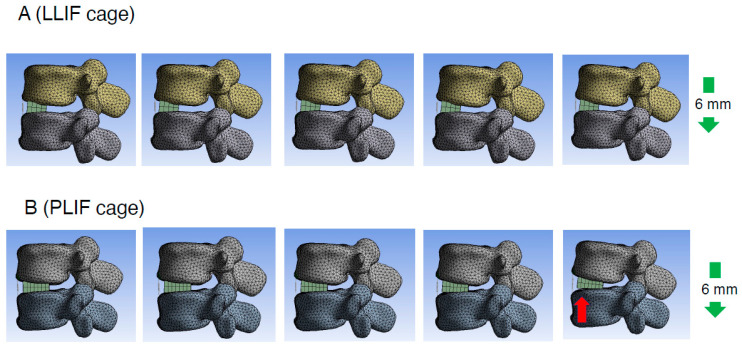
Representative cases of the cantilever technique model (spinous process displacement model). Consecutive images illustrating the spinous process displacement in the cantilever technique model. The right side depicts the later phase of the displacement process. It was observed that with the use of a PLIF cage, the posterior edge of the cage became a hinge and the gap between the cage and endplate became more pronounced (red arrow) in the later phases. LLIF indicates lateral lumbar interbody fusion; PLIF, posterior lumbar interbody fusion.

**Figure 7:**
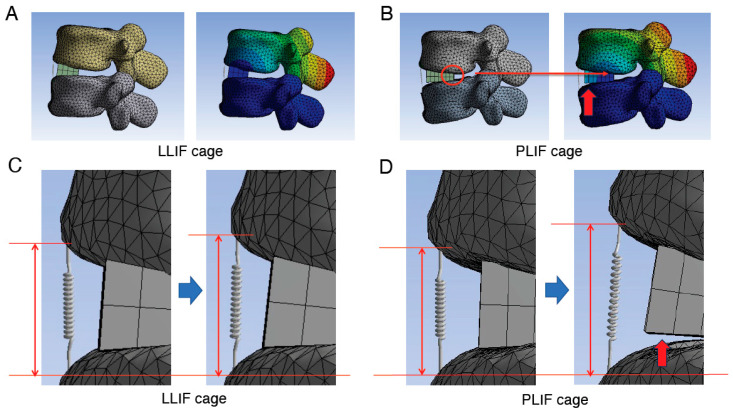
Detailed images of the assessment in the cantilever technique model. (**A**,**B**), Assessment of the location of the rotation center when using LLIF cage (**A**) or PLIF cage (**B**) in the cantilever technique model. When the PLIF cage is used, the longer A-P length of the cage causes the posterior edge of the cage to become the hinge (red circle) and the center of rotation to be located near this area, which in turn applies a greater load to the ALL due to the principle of leverage. (**C**,**D**), Enlarged images of the site of spring before (left side images of **C**,**D**) and after (right side images of **C**,**D**) correction procedure. The degree of spring elongation is more obvious when the PLIF cage was used. In addition, in later phases, the posterior edge of the cage acts as a hinge, leading to an observable gap between the cage and the endplate (indicated by red arrows in panels (**B**,**D**)). ALL indicates anterior longitudinal ligament; A-P, anterior–posterior; LLIF, lateral lumbar interbody fusion; PLIF, posterior lumbar interbody fusion.

To assess the direct burden on the ALL in the cantilever technique model (spinous process displacement model), the elongation degree of the spring set at the location of the ALL was evaluated. The average spring elongation in the PLIF and LLIF groups was 2.7 ± 0.3 mm and 1.7 ± 0.5 mm, respectively (average ± SD). The elongation degree in the PLIF group was significantly larger than that in the LLIF group (*p* = 0.006, Cohen’s d = 2.33, Power (1−β) = 0.956) (Figure 4C and Table 1). The values for Cohen’s d and Power (1−β) were large enough to ensure the magnitude of the significant difference and the validity of the test method. Therefore, in the cantilever technique model, the direct burden on the ALL in the PLIF group was considered significantly larger than that in the LLIF group. It is notable that this difference was statistically significant only in the cantilever technique model, not in the PS compression model (Figure 4A,C and Table 1). Representative cases of the cantilever technique model are presented in Figure 6 and Figure 7. As shown in Figure 6 and Figure 7, the posterior edge of the PLIF cage became a hinge and the gap between the cage and endplate became noticeable in the later phases, owing to its longer A-P length (Figure 6 and Figure 7).

### 3.4. Assessment of the Rotation Center Location in the Cantilever Technique Model (Spinous Process Displacement Model)

To assess the indirect burden on the ALL in the cantilever technique model (spinous process displacement model), the location of the rotation center was also evaluated. The average location of the rotation center in the PLIF group was 0.59 ± 0.06, while that in the LLIF group was 0.43 ± 0.10 (average ± SD). The location of the rotation center in the PLIF group was located significantly more posterior than that in the LLIF group (*p* = 0.013, Cohen’s d = 2.00, Power (1−β) = 0.891) (Figure 4D and Table 1). The values for Cohen’s d and Power (1−β) were large enough to ensure the magnitude of the significant difference and the validity of the test method. Therefore, the indirect burden on the ALL in the PLIF group in the cantilever technique model was significantly larger than that in the LLIF group.

## 4. Discussion

As mentioned above, corrective surgery for ASD has made significant progress in various ways [10,11,12,13,14], but several unresolved issues remain. One particularly noteworthy issue is the occasional rupture of the ALL during posterior corrective procedures for ASD (Figure 1). ALL rupture increases the risk of pseudoarthrosis, so its prevention is essentially important [14,15,16]. While using some kind of instrumentation during anterior cage placement may prevent ALL rupture during posterior correction, it is not a desirable option because it makes it impossible to achieve further lumbar lordosis through additional posterior osteotomy. Therefore, it is important to identify the cause of ALL rupture during posterior correction so that preventive measures can be taken. However, the exact mechanism of ALL rupture during posterior corrective surgery remains unclear. ALL rupture during posterior correction is thought to be a multifactorial phenomenon, and we have conducted various analyses to date. We have previously reported that one of the causes is likely to be the use of a cage with an insufficient lordotic angle that is inappropriate for the degree of posterior osteotomy [32].

On the other hand, in recent years, the benefits of LLIF have become widely known, and LLIF is increasingly being used in corrective surgery for ASD [10,12,13]. It is notable that ALL rupture during posterior corrective procedure is a phenomenon that was infrequent prior to the introduction of LLIF. Before the widespread use of LLIF, interbody fusion was exclusively performed using PLIF or TLIF in corrective surgery for ASD [10,12,13]. Therefore, it is possible that the use of LLIF in corrective surgery for ASD may be the cause of the aforementioned ALL rupture. On the other hand, with advancements in ASD corrective capabilities in recent years, it remains unclear whether ALL rupture during posterior correction is specifically associated with the use of LLIF or reflects various improvements in corrective capabilities [10,12,13]. To the best of our knowledge, no previous papers have been published on this subject. This issue is extremely important because if the combined use of LLIF appears to be the cause of ALL rupture, we would conclude that it would be wise to reconsider our surgical plan itself. To investigate this, we conducted FEA to explore the mechanisms underlying ALL rupture using two different types of intervertebral cages, LLIF and PLIF cages. Additionally, we developed two different types of FEA models simulating compression procedures for common lumbar degenerative diseases and posterior corrective procedures for ASD.

Our previous research indicated that utilizing intervertebral cages with adequate lordosis, tailored to the degree of osteotomy, may mitigate ALL burden compared to less lordotic cages [32]. Therefore, both LLIF and PLIF cages employed in this study were configured with a 12-degree lordotic angle to maintain consistency with optimal lordosis requirements.

Maruo et al. reported a 22% incidence of ALL injury in patients undergoing posterior correction procedures for ASD following prior LLIF. They conducted multivariate analyses identifying XLIF and preexisting osteoporotic vertebral fractures as risk factors for ALL injury [14]. However, it is plausible that some ALL injuries occurred during the preceding LLIF procedure but were only detected during the subsequent posterior correction. Thus, clinical studies aiming to identify risk factors for ALL injury during posterior correction procedures may be confounded by cases where the ALL was injured during prior LLIF, leading to potential misinterpretations.

Given these limitations, clinical case series may not accurately pinpoint risk factors for ALL injury during posterior correction after LLIF. Moreover, the etiology of ALL injury during posterior correction procedures is likely multifactorial. Therefore, FEA represents a valuable approach to systematically analyze the mechanisms of ALL injury in such procedures.

ALL injury during posterior correction procedures is observed primarily in ASD corrections, distinct from fusion surgeries for common degenerative lumbar diseases. To investigate this phenomenon, we developed two different types of FEA models: the PS compression model and the cantilever technique model. These models simulate fusion surgeries for common lumbar degenerative diseases and corrective procedures for ASD, respectively.

In this study, we observed that both the direct and indirect burdens on the ALL were larger in the PLIF group compared to those in the LLIF group. Importantly, the difference in the direct burden on the ALL was statistically significant only in the cantilever technique model, not in the PS compression model (Figure 4A,C and Table 1).

During applications of substantial force, such as those required to correct kyphosis following grade 2 PCO, the posterior edge of the PLIF cage acts as a fulcrum. Our analysis indicates that the longer A-P length of the PLIF cage positions its posterior edge as a hinge, establishing the center of rotation proximate to this region. Consequently, this configuration applies increased load to the ALL, following the principle of leverage (Figure 7B).

Although there are a number of papers discussing the effect of various interbody cage geometry on bone union or cage sinking, etc. [35], few have discussed their effect on susceptibility to ALL rupture. Maruo et al. mentioned the possibility that differences in the cage geometry used when performing LLIF may lead to differences in the susceptibility to ALL rupture [14], but to our knowledge, there are no papers that have investigated whether differences in the geometry of the LLIF cage and PLIF cage lead to differences in the susceptibility to ALL rupture.

Considering the results of our current study, ALL rupture observed during posterior correction procedures appears to correlate more with the magnitude of correction force rather than being solely influenced by the geometry of the LLIF cage.

This study has several limitations. First, there are differences between PLIF and LLIF regarding disc cleaning methods and other procedural aspects. Hence, based on this study’s findings, it is reasonable to conclude that the geometry of the LLIF cage is not the sole cause of ALL rupture. Second, the actual ALL exhibits non-linear elastic properties. In this study, ALL was modeled as a linear spring; however, it has non-linear elastic properties in reality, which were not considered. However, in this study, we aimed to conduct a comparative analysis between two groups using different types of intervertebral cages, rather than determining absolute rupture strength values. Therefore, we opted to use a spring model to evaluate the burden on the ALL to simplify the experimental model. Third, bone quality (e.g., osteoporosis) was not taken into account in this study, which limits clinical applicability and generalizability. In addition, only a single-level analysis was performed; in multi-level deformity corrections, loading patterns may differ. We performed a single-level analysis to simplify the FEA model as a preliminary step in the current study, and in the next phase, we plan to conduct multi-level analyses to better approximate clinical scenarios and retrospective studies using large-scale clinical data.

## 5. Conclusions

The results of this analysis suggest that when the PLIF cage is used, the longer A-P length of the cage causes the posterior edge to act as a hinge, with the center of rotation located proximal to this area. This configuration applies a greater load to the ALL due to the principle of leverage. Taken together, this biomechanical analysis using FEA concluded that ALL rupture during posterior correction is thought to be primarily associated with increased correction ability rather than solely attributable to the geometry of the LLIF cage. On the other hand, this study has limitations as mentioned above, so that the conclusions would be further strengthened by further research that supports the findings with multi-level analyses and retrospective studies using large-scale clinical data.

## Figures and Tables

**Table 1:** Summary of statistical analysis results.

			Average ±SD	*p*	Cohen’s d	Power (1−β)
**cantilever technique model**	**spring elongation**	**LLIF**	1.7 ± 0.5	0.006 *	2.33	0.956
		**PLIF**	2.7 ± 0.3			
	**rotation center**	**LLIF**	0.43 ± 0.10	0.013 *	2.00	0.891
		**PLIF**	0.59 ± 0.06			
**PS compression model**	**spring elongation**	**LLIF**	1.9 ± 0.2	0.773	0.19	0.085
		**PLIF**	2.0 ± 0.4			
	**rotation center**	**LLIF**	0.44 ± 0.06	0.008 *	2.23	0.941
		**PLIF**	0.63 ± 0.10			

LLIF indicates lateral lumbar interbody fusion; PLIF, posterior lumbar interbody fusion; SD, standard deviation. * indicate statistically significant differences.

## Data Availability

The datasets used and/or analyzed in this study are available from the corresponding author on reasonable request.
