# Correction: Imai et al. Is Anterior Longitudinal Ligament Rupture During Posterior Corrective Surgery for Adult Spinal Deformity a Phenomenon Unique to When Combined with Lateral Lumbar Interbody Fusion? -Finite Element Analysis with Comparison to When Combined with Posterior Lumbar Interbody Fusion-. *J. Clin. Med.* 2025, *14*, 7460

**DOI:** 10.3390/jcm15020728

**Published:** 2026-01-16

**Authors:** Takaya Imai, Hiroki Takeda, Yuichiro Abe, Koutaro Kageshima, Yuki Akaike, Soya Kawabata, Nobuyuki Fujita, Shinjiro Kaneko

**Affiliations:** 1 Department of Spine and Spinal Cord Surgery, School of Medicine, Fujita Health University, 1-98 Dengakugakubo, Kutsukake-cho, Toyoake 470-1192, Aichi, Japan; 2 Department of Orthopedic Surgery, School of Medicine, Fujita Health University, Toyoake 470-1192, Aichi, Japan

**Title/Heading Correction**

There was a spelling mistake in the original publication [1]. A correction has been made to “Is Anterior Longitudinal Ligament Rupture During Posterior Corrective Surgery for Adult Spinal Deformity a Phenomenon Unique to When Combined with Lateral Lumbar Interbody Fusion? -Finite Element Analysis with Comparison to When Combined with Posterior Lumbar Interbody Fusion-” and “2.1. Establishment of the Three-Dimensional (3D) Finite Element (FE) Model of the Lumbar Spine”.

**Figure 1** **Legend**

In the original publication [1], the following sentence was missing: “Reproduced with permission from Takeda, H.; Abe, Y.; Imai, T.; Rashid, M.Z.M.; Ikeda, D.; Kawabata, S.; Nagai, S.; Hachiya, K.; Fujita, N.; Kaneko, S. Elucidation of the mechanism of occasional anterior longitudinal ligament rupture with posterior correction procedure for adult spinal deformity using LLIF-finite element analysis of the impact of the lordotic angle of intervertebral LLIF cage. *Medicina* 2023, *59*, 1569; published by MDPI, 2023. [32].”

The correct legend appears below.

**Figure 1:**
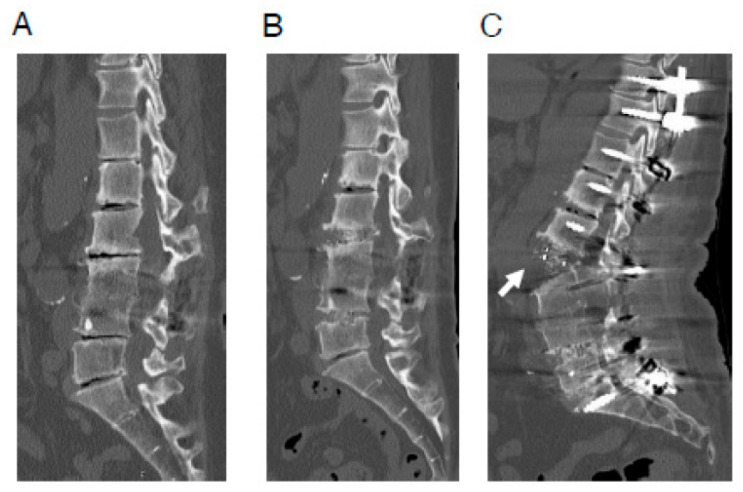
A representative case of ALL rupture during posterior correction procedure. A 64-year-old woman with adult spinal deformity, who had undergone postero-lateral fusion at the L3/4 level previously at another hospital. (**A**) Preoperative CT image. (**B**) CT image after OLIF (L2/3·L4/5). (**C**) CT image after posterior correction procedure. In this case, surgery was performed using less lordotic (6°) OLIF cages, which were most lordotic cage available at the time. Anterior longitudinal ligament rupture (white arrow) was observed after the posterior correction procedure. ALL—anterior longitudinal ligament; CT—computed tomography; LLIF—lateral lumbar interbody fusion; OLIF—oblique lateral lumbar interbody fusion. Reproduced with permission from Takeda, H.; Abe, Y.; Imai, T.; Rashid, M.Z.M.; Ikeda, D.; Kawabata, S.; Nagai, S.; Hachiya, K.; Fujita, N.; Kaneko, S. Elucidation of the mechanism of occasional anterior longitudinal ligament rupture with posterior correction procedure for adult spinal deformity using LLIF-finite element analysis of the impact of the lordotic angle of intervertebral LLIF cage. *Medicina* 2023, *59*, 1569; published by MDPI, 2023. [32].

The authors state that the scientific conclusions are unaffected. This correction was approved by the Academic Editor. The original publication has also been updated.

## References

[1] Imai T., Takeda H., Abe Y., Kageshima K., Akaike Y., Kawabata S., Fujita N., Kaneko S. (2025). Is Anterior Longitudinal Ligament Rupture During Posterior Corrective Surgery for Adult Spinal Deformity a Phenomenon Unique to When Combined with Lateral Lumbar Interbody Fusion? -Finite Element Analysis with Comparison to When Combined with Posterior Lumbar Interbody Fusion-. J. Clin. Med..

